# Three agonist antibodies in combination with high-dose IL-2 eradicate orthotopic kidney cancer in mice

**DOI:** 10.1186/1479-5876-8-42

**Published:** 2010-04-28

**Authors:** Jennifer A Westwood, Phillip K Darcy, Preethi Mayura Guru, Janelle Sharkey, Hollie J Pegram, Sally M Amos, Mark J Smyth, Michael H Kershaw

**Affiliations:** 1 Cancer Immunology Research Program, Peter MacCallum Cancer Centre, Melbourne, Australia; 2 Department of Pathology, University of Melbourne, Australia; 3 Department of Immunology, Monash University, Clayton, Australia

## Abstract

**Background:**

Combination immunotherapies can be effective against subcutaneous tumors in mice but the effect against orthotopic malignant disease is less well characterized. In particular, a combination of three agonist antibodies, termed Tri-mAb, consisting of anti-DR5, anti-CD40 and anti-CD137 has previously been demonstrated to eradicate a large proportion of subcutaneous renal cell carcinoma (Renca) tumors (75% long-term survival), but the effect against orthotopic disease is not known.

**Purpose:**

To determine the relative response of orthotopic tumors, we inoculated Renca into the kidney followed by treatment with Tri-mAb.

**Results:**

We found that orthotopic tumors responded much less to treatment (~13% survival), but a significant improvement in survival was achieved through the addition of IL-2 to the treatment regimen (55% survival). All three agonist antibodies and high dose IL-2, 100,000 IU for up to six doses, were required. CD8^+ ^T cells were also required for optimal anti-tumor responses. Coadministration of IL-2 led to enhanced T cell activity as demonstrated by an increased frequency of IFN-gamma-producing T cells in tumor-draining lymph nodes, which may have contributed to the observed improvement of therapy against kidney tumors.

**Implications:**

Responses of subcutaneous tumors to immunotherapy do not necessarily reflect how orthotopic tumors respond. The use of combination immunotherapy stimulating multiple facets of immunity and including cytokine support for T cells can induce effective anti-tumor responses against orthotopic and metastatic tumors.

## Introduction

Immunotherapies involving combinations of various immunomodulating agents are demonstrating considerable promise for the treatment of cancer. In particular, the use of agents that together stimulate multiple immune components can mediate regression of established tumors. Important steps to achieve robust anti-tumor immunity include tumor antigen release, optimal antigen presentation to specific T cells and costimulation of T cells resulting in optimal activation and expansion of tumor-specific T cells.

Monoclonal antibodies (mAb) targeting death receptors expressed on a range of transformed cells [1] can mediate apoptosis of a proportion of tumor cells leading to induction of tumor-specific T cells and inhibition of tumor growth in preclinical mouse models[2]. An agonistic antibody targeting CD40 expressed on antigen presenting cells has been demonstrated to lead to activation of APCs and the generation of CTL and eradication of lymphoma in mice[3]. Triggering the costimulatory molecule CD137 (4-1BB) expressed on activated T cells [4] has been demonstrated to lead to increases in T cell numbers and activation [5,6]. Agonistic antibodies specific for CD137 can inhibit tumor growth in mice [7].

However, this use of single immunomodulators against established disease has been of limited effect in both preclinical and early phase clinical trials [8-10]. The use of immunomodulating agents in combination with chemotherapy is demonstrating promise, and drug-induced tumor apoptosis and immune-potentiation are thought to play a role in therapy using combined agents [11,12].

Combinations of immune agonistic antibodies have also demonstrated effectiveness against tumors of various histologies when implanted subcutaneously. A combination of three antibodies targeting DR5, CD40 and CD137, termed Tri-mAb, was able to induce complete regression of syngeneic breast and kidney cancers located subcutaneously [13]. In another study using this combination approach, NKT cell glycolipid ligands were demonstrated to be able to substitute for CD40 ligation and induce tumor regression [14]. A subsequent study demonstrated that the inclusion of IL-21 in the treatment schedule could enhance the efficacy of Tri-mAb therapy against subcutaneous disease and small metastases [15].

Since tumor growth and responses can vary depending on size and anatomical location, and established orthotopic metastatic cancer is considered more difficult to treat than subcutaneous disease, in the current study we sought to determine the effect of Tri-mAb against established orthotopic and metastatic renal cell carcinoma without nephrectomy and ascertain if treatment could be optimized using cytokine support.

## Materials and methods

### Cell lines and mice

Renca is a kidney cancer cell line of BALB/c mice [16]. This tumor cell line was maintained at 37°C and 5% CO_2 _in RPMI medium, supplemented with 10% heat-inactivated fetal calf serum (FCS) (Moregate Biotech, Bulimba, QLD, Australia), 2 mM glutamine (JRH Biosciences, Brooklyn, VIC, Australia), 100 U/ml penicillin, and 100 μg/ml streptomycin (both from Sigma, Castle Hill, NSW, Australia).

BALB/c mice were purchased from The Walter and Eliza Hall Institute of Medical Research, Melbourne, Australia, and from Animal Resource Centre, Perth, Western Australia. They were housed in specific pathogen free conditions. Mice of 6 to 20 weeks of age were used in experiments, and experiments were performed according to The Peter MacCallum Cancer Centre Animal Experimentation Ethics Committee guidelines.

### Tumor growth in mice

BALB/c mice were inoculated subcapsule into the kidney with 1 × 10^5 ^Renca cells. Treatment started 10 - 11 days later, after randomization of mice into groups. Tri-mAb consisted of a mixture of MD5.1 (anti-death receptor-5, DR5), FGK-45 (anti-CD40) and 3H3 (anti-4-1BB) in equal proportions. Each antibody was determined to be endotoxin free by LAL test. Different batches of the individual mAbs of Tri-mAb were titrated in this study so that 50-80% tumor regression of s.c. tumors was achieved, as some batches of antibodies were toxic at the highest dose of 100 μg each mAb per dose. Each dose of Tri-mAb was injected intra-peritoneally every 3 - 4 days for a total of 3 doses. Other groups additionally received 100,000 IU recombinant human IL-2 (NCI Preclinical Repository, Frederick, MD, USA) in 200 μl PBS i.p. every day for up to 6 doses or until mice could not tolerate any more. Some groups received PBS or control MAC4 rat IgG2a and UC8-1B9 hamster IgG instead of Tri-mAb, at 100 μg each mAb/200 μl/dose intra-peritoneally every 3 - 4 days for a total of 3 doses. Tumor progression was determined by survival of mice, which was defined as time until mice became moribund, at which point they were euthanized. Subcutaneous tumors were established by injection of 2 × 10^5 ^Renca cells in 100 μl PBS into the abdominal area of mice after fur in that area was trimmed. In some experiments CD8^+ ^T cells were depleted using the mAb, clone 53.6.72, administered 200 μg 1 day prior to tumor injection and 100 μg on the day of tumor injection followed by twice weekly injections of 100 μg for 24 days.

### Intracellular IFN-γ assay

Mice were injected with 2 × 10^5 ^Renca s.c. into the upper foot. Some groups received Tri-mAb on days 10 and 13, and/or IL-2 at the dose of 100,000 IU/200 μl i.p. on days 10, 11 and 13. Control mice received MAC4 plus UC8-1B9 i.p. On day 14, mice from each group were culled and popliteal lymph node cells were harvested, teased between forceps, filtered through a 70 μm filter, and individually stimulated on anti-CD3 coated plates (0.13 μg per well). 1 × 10^6 ^cells were placed in 0.5 ml media in a 48-well plate. The plates were incubated for 18 hours at 37°C in 5% CO_2_, and GolgiStop (BD Biosciences, San Jose, CA) was added for the last 12 hours. Cells were harvested the next day and stained and assessed by flow cytometry for intracellular IFNγ using a Cytofix/Cytoperm Plus Fixation/Permeabilization kit (BC Biosciences Cat # 554715), following the manufacturer's directions. Antibodies used in this study were CD8α-FITC clone 53-6.7 (BD), TCRβ-APC clone H57.597 (BD), and IFNγ-PE clone XMG1.2 (BD). Cells were analyzed by flow cytometry using a FACS Calibur (BD).

### Statistical analysis

Statistical analysis was performed with StatsDirect software using Log rank analysis for comparing mouse survival data, and a Mann-Whitney Test for comparing cytokine secretion from different treatment groups. Data producing a P value of < 0.05 was considered to be significant.

## Results

### Tri-mAb therapy is less effective against tumors inoculated into the kidney

Previous studies have demonstrated that administration of Tri-mAb (anti-DR5 + anti-CD40 + anti-CD137) to mice bearing subcutaneous Renca tumors regularly resulted in complete regression of 50-80% (mean 70.8% ± 6.4) of tumors [13]. In order to determine if this degree of effectiveness could be achieved against orthotopic metastatic disease, we inoculated Renca directly into the kidney and determined the effect of Tri-mAb on mouse survival compared to mice bearing subcutaneous tumors. Treatment with Tri-mAb began 10 days after inoculation of Renca tumor cells, by which time s.c. tumors were approximately 30 mm^2 ^in size, and primary kidney tumors were approximately 10 mm^2^. Micrometastases were also present at day 10 in the lungs of mice bearing kidney tumors.

**Figure 1:**
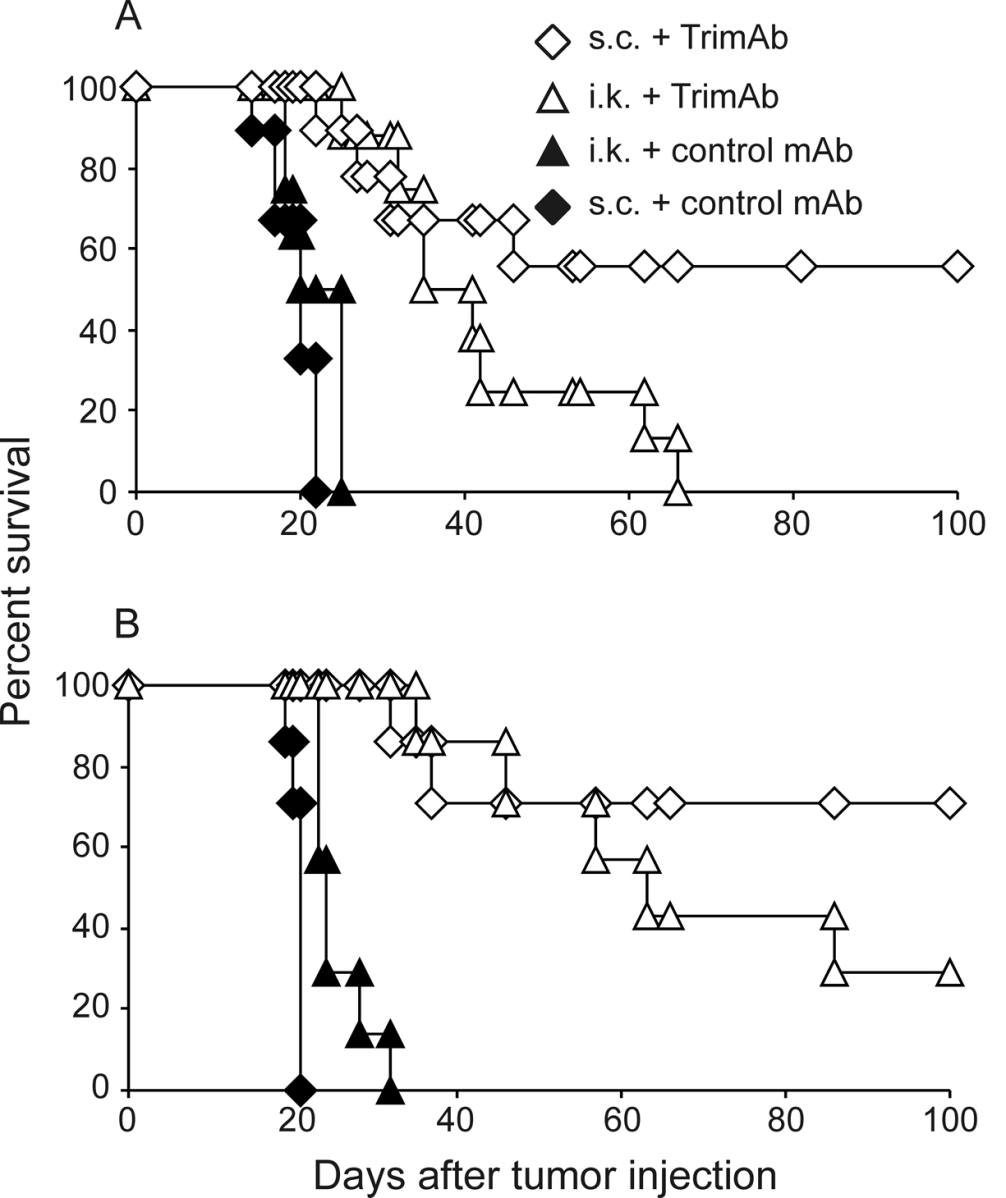
**Tri-mAb is less effective against orthotopic kidney cancer than it is against subcutaneous tumors**. Mice were injected with Renca cells either subcutaneously (s.c.) (2 × 10^5^) or intrakidney (i.k.) (1 × 10^5^). On day 10 after tumor injection, some groups of mice received Tri-mAb treatment consisting of 100 μg each of anti-DR5, anti-CD40 and anti-CD137 on days 10, 13 and 17. Other groups of mice received control treatment consisting of isotype control antibodies (MAC4, rat IgG2a; UC8-1B9, hamster IgG) or were left non-treated (NT), and survival monitored. Two representative experiments of three are shown A and B. Control refers to non-treated in panel A and MAC4 rat IgG2a and UC8-1B9 isotype controls for panel B. Survival of s.c. tumor-bearing mice greater than mice with kidney tumors, P2 = 0.034 for pooled data from A and B, Log-rank Test. (s.c. = subcutaneous tumor; i.k. = intrakidney tumor).

Non-treated mice, and those treated with isotype control mAb, died before day 30 after tumor inoculation as demonstrated in two independent experiments (Figure 1). Mice bearing subcutaneous tumors survived significantly longer than control mice, with 55% to 100% (mean 74.3% ± 13.6) of Tri-mAb-treated mice surviving long-term. The survival of Tri-mAb-treated mice bearing kidney tumors was significantly longer than control mice (P2 < 0.0001, Log-rank test), but significantly shorter than mice with subcutaneous tumors that received Tri-mAb (P2 = 0.034, Log-rank test for pooled data) (Figure 1). Mice inoculated with subcutaneous tumors were culled when tumors reached the ethically maximum ethically-permitted size of 150 mm^2^. No metastases were present in mice bearing subcutaneous tumors. Mice inoculated with kidney tumors were culled when signs of stress became evident, as indicated by lethargy and hunched, ruffled appearance. Mice that died in groups inoculated with kidney tumors all had large primary kidney tumors and/or abdominal and lung metastases, as seen at necropsy. Tri-mAb treatment of kidney tumor-bearing mice generally resulted in 0% to 30% long-term survivors, but it should be noted that in one experiment 75% of mice eradicated kidney tumors following Tri-mAb treatment (data not shown). It is not entirely clear why a large proportion of mice survived in that experiment, although Tri-mAb treatment was associated with considerable toxicity (weight loss and lethargy) in that case, suggesting one or more antibodies in that batch of Tri-mAb was unusually active.

### The effectiveness of Tri-mAb against orthotopic kidney cancer is enhanced by coadministration of high-dose IL-2

**Figure 2:**
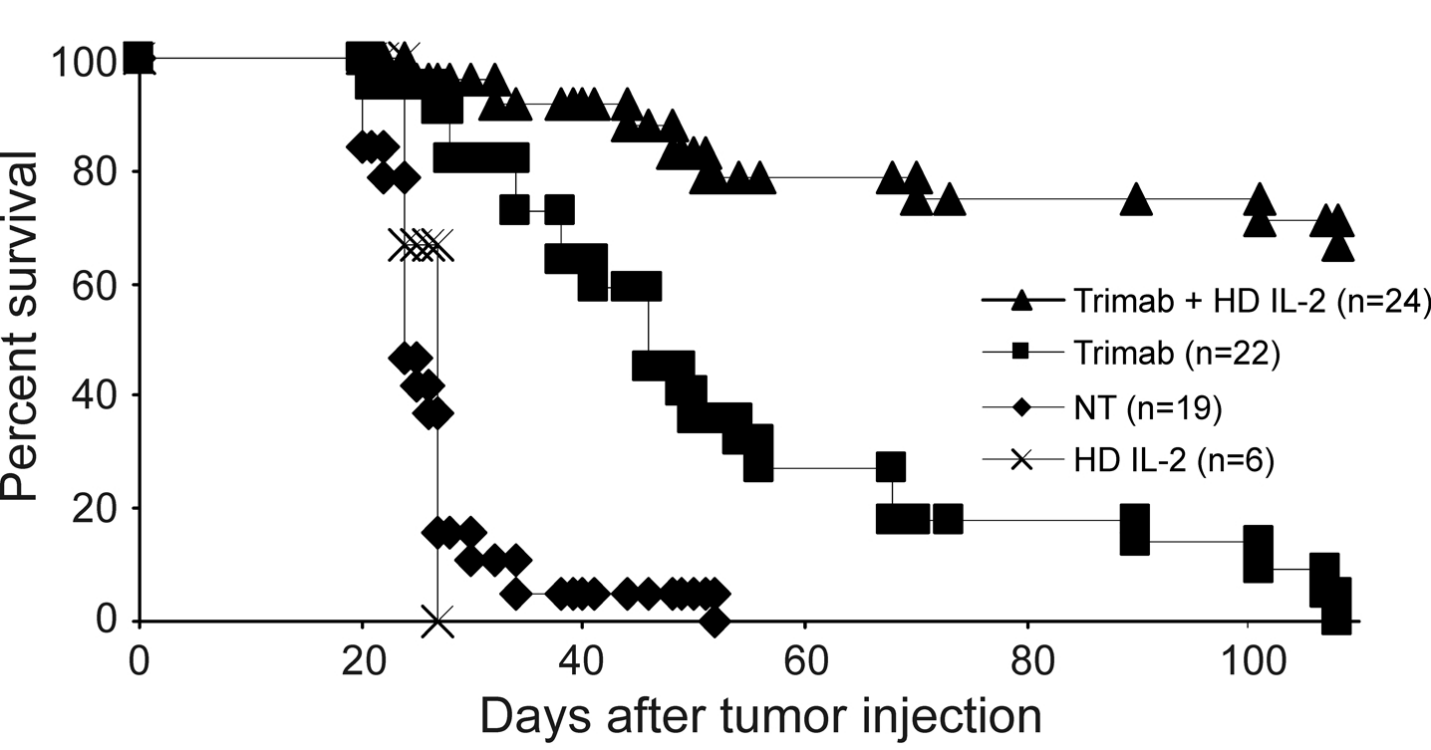
**Coadministration of IL-2 enhances the survival of mice bearing orthotopic kidney cancer**. Groups of mice bearing 10-day established kidney tumors were treated with either Tri-mAb alone (days 10, 14 and 18), IL-2 alone (10^5 ^IU i.p. daily for 4 to 6 days) or the combination of Tri-mAb + IL-2. Other groups were non-treated (NT). Numbers in parentheses depict the number of mice per group. Pooled results of 4 experiments are shown. (P2 < 0.0001 for Tri-mAb + HD IL-2 vs Tri-mAb alone, Log-rank Test). (NT = non-treated, HD = high dose).

Since CD8^+ ^T cells were previously demonstrated to play a crucial role in Tri-mAb therapy [13], we reasoned that coadministration of the T cell growth factor, IL-2, may enhance Tri-mAb therapy of orthotopic kidney cancer. When Tri-mAb alone was used against 10-day-established kidney cancer, a significant survival advantage of mice was achieved P2 < 0.0001) but no mice survived long-term (Figure 2). When high dose IL-2 was added to the treatment regimen, a further significant survival advantage over treatment with Tri-mAb alone was gained (P2 < 0.0001), with approximately 65% now surviving long-term beyond day 110.

In the experiments described above, we used high dose IL-2 at 100,000 IU administered intraperitoneally daily for 5 days. To determine if a similar enhancement of therapy could be achieved with lower doses of IL-2, we treated tumor-bearing mice with Tri-mAb in combination with low dose (10,000 IU) or medium dose (50,000 IU) or high dose (100,000 IU) IL-2 daily for 5 days and monitored survival.

**Figure 3:**
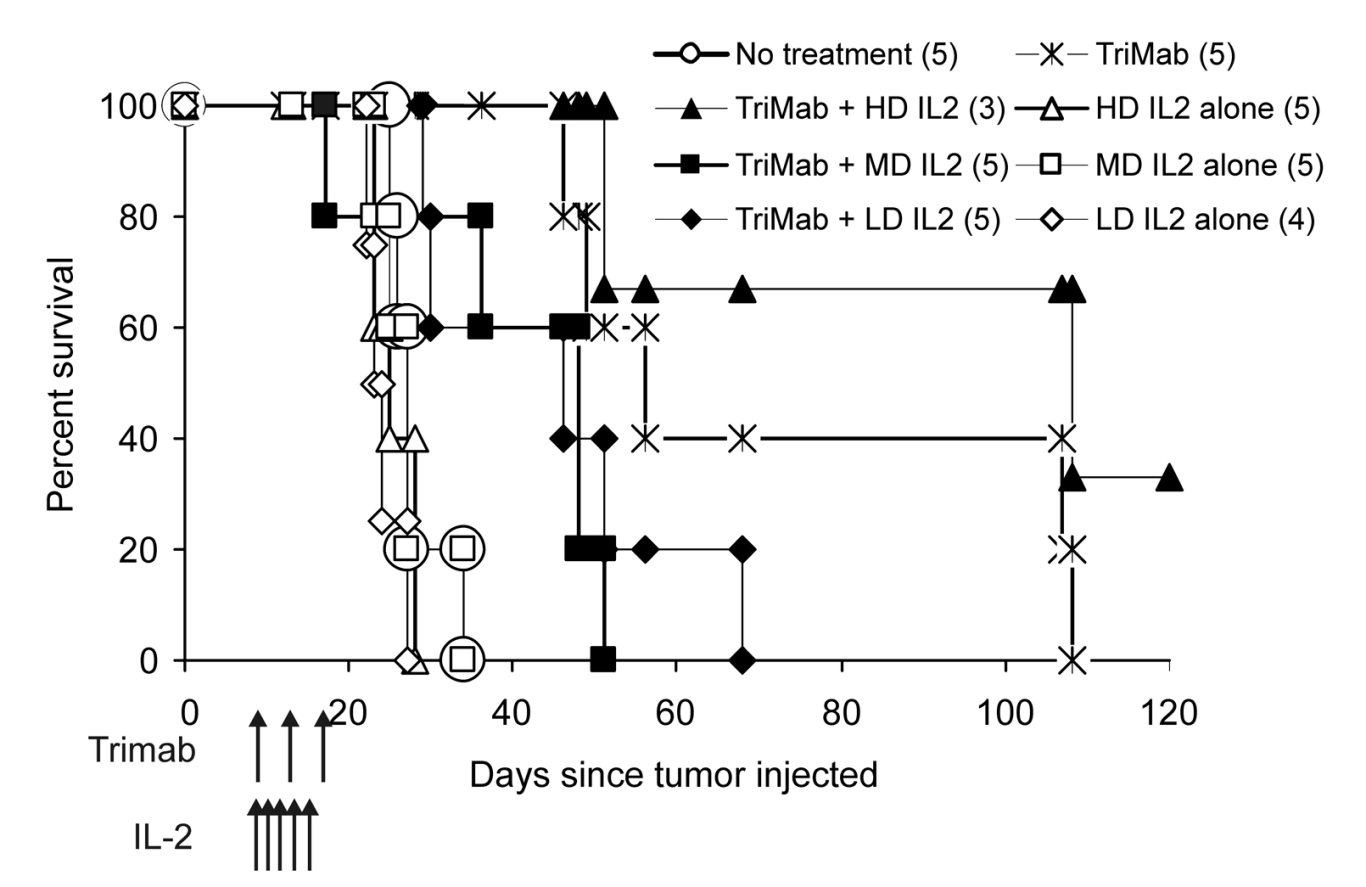
**Large doses of IL-2 are necessary for optimal anti-tumor activity**. Mice with established orthotopic disease were treated with Tri-mAb alone or in combination with various dose levels of IL-2 delivered in five doses as indicated. HD: high dose (10^5 ^IU); MD: medium dose (5 × 10^4 ^IU); LD: low dose (10^4 ^IU). Numbers in parentheses depict number of mice per group. Experiment performed once, P < 0.018 for high-dose IL-2 vs lower doses (Log-rank test).

Mice receiving Tri-mAb with low or medium dose IL-2 did not survive longer than mice receiving Tri-mAb alone (Figure 3). Only with coadministration of high dose IL-2 was the survival of mice significantly increased beyond those that received Tri-mAb alone. The enhanced survival was not due to high dose IL-2 alone since mice receiving IL-2 alone did not survive longer than non-treated mice.

### All three antibodies are necessary for optimal anti-tumor effects and treatment induces anti-tumor immunological memory

**Figure 4:**
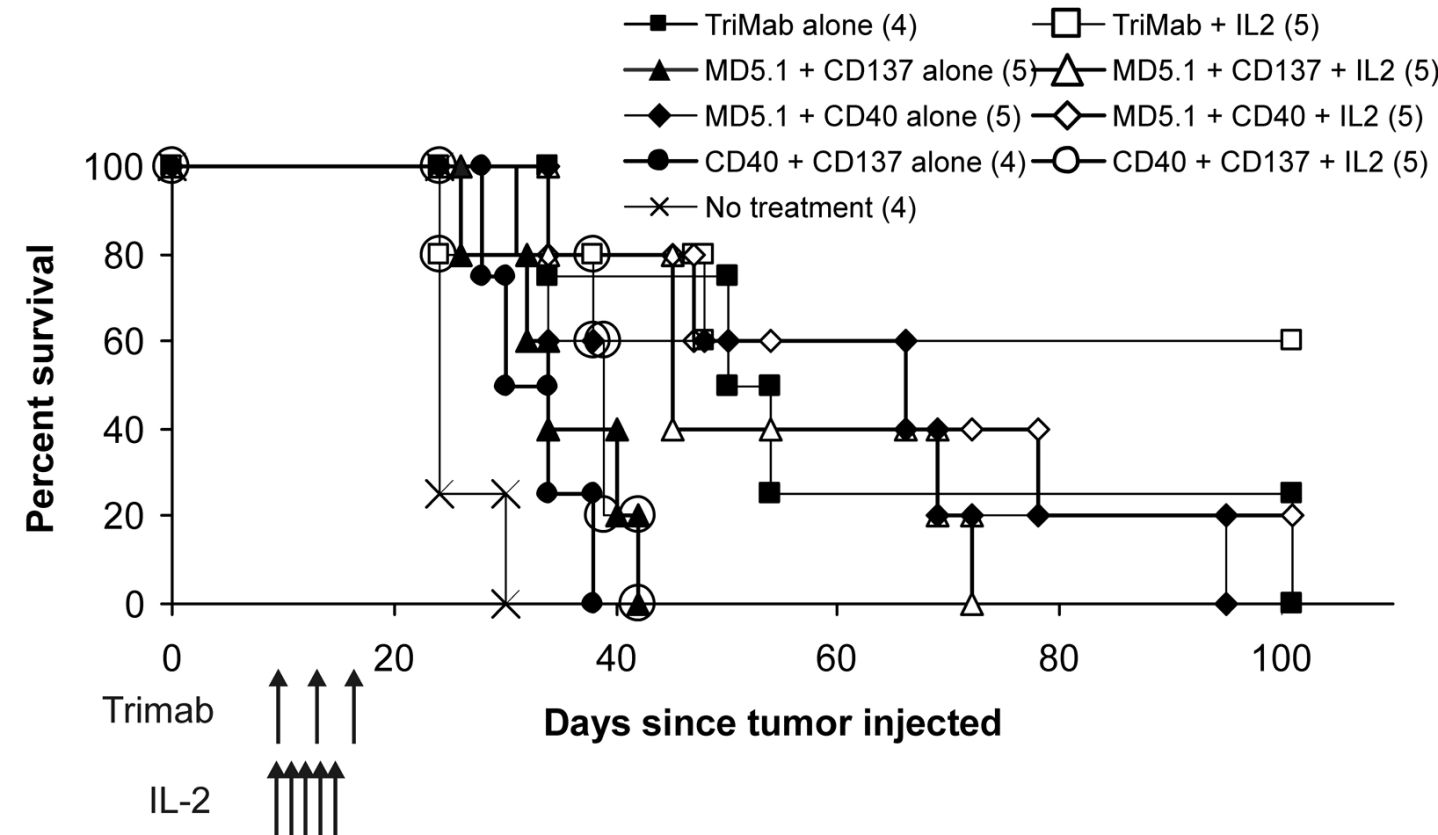
**All three agonist antibodies are necessary in combination with IL-2 for optimal anti-tumor effects**. Following intrakidney injection of Renca cells, mice received the treatments listed as indicated by arrows. Some mice received the complete treatment of Tri-mAb + IL-2, whereas other groups received various pairs of antibodies as listed either alone or in combination with IL-2. Some mice received no treatment to act as controls. Numbers in parentheses depict number of mice per group. Results from a single experiment, P < 0.05 for three antibodies + IL-2 vs two antibodies + IL-2 (Log-rank test), except for the comparison between Tri-mAb + IL-2 and MD5.1/CD40 + IL-2 which tended towards significance at P = 0.079.

To determine if all three antibodies were necessary for the optimal enhancement of therapy by IL-2, groups of mice were treated with each combination of two antibodies and IL-2 and survival compared to mice receiving three antibodies and IL-2. All treatment groups survived longer than non-treated mice, but the largest proportion of long-term surviving mice was in the group receiving all three antibodies plus IL-2 (Figure 4).

Previous studies have shown that subcutaneous tumor-bearing mice surviving long-term after Tri-mAb therapy are resistant to tumor rechallenge, demonstrating immunological memory formation against tumor-associated antigens [13]. To determine if memory was similarly invoked in mice bearing orthotopic kidney cancer following the inclusion of IL-2 in the treatment regimen, we rechallenged surviving mice with Renca (2 × 10^5^, subcutaneously).

**Figure 5:**
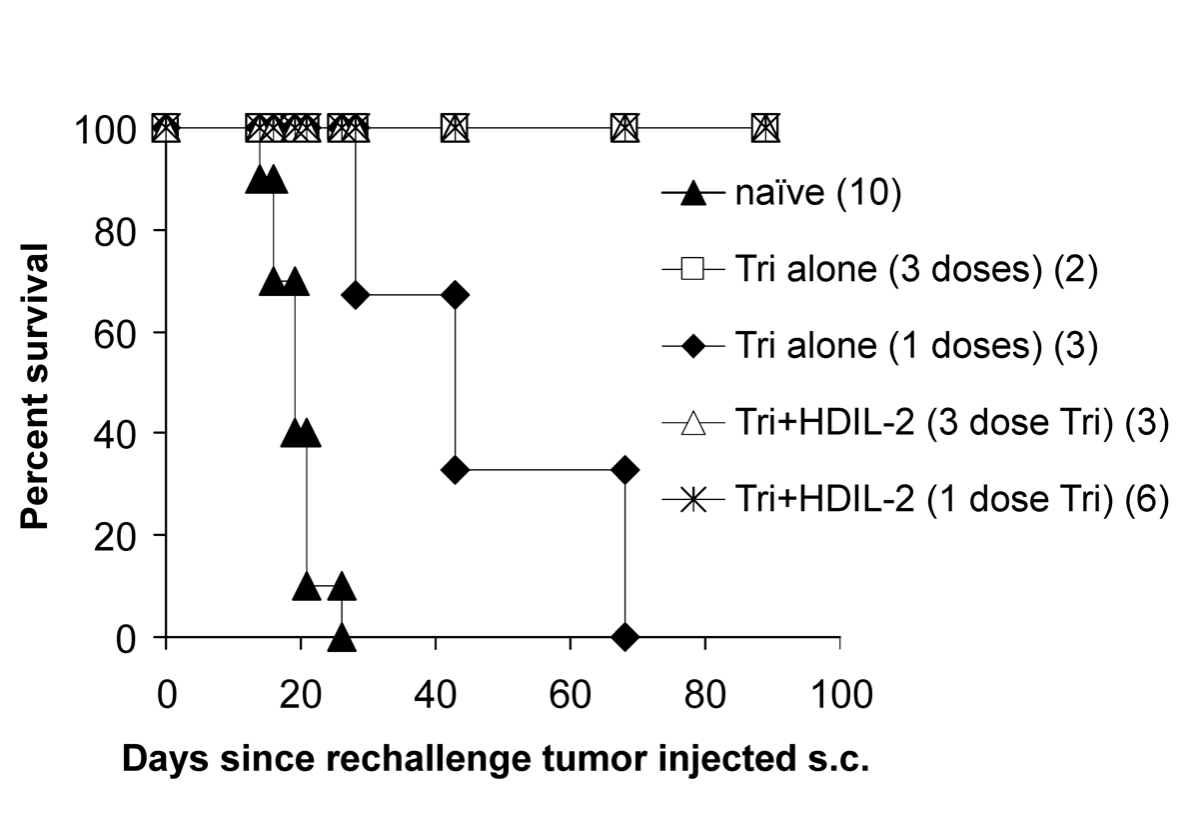
**Tri-mAb in combination with IL-2 induced anti-tumor immunological memory**. Long-term surviving mice were rechallenged with a subcutaneous injection of Renca (2 × 10^5 ^cells) and survival monitored. Mice were derived from groups of kidney tumor-bearing mice that received either 3 doses of Tri-mAb alone or in combination with IL-2. Some mice came from groups receiving only one dose of Tri-mAb with or without IL-2. A group of naive mice was also injected subcutaneously with Renca as controls for tumor growth in the absence of immunological memory. Numbers in parentheses depict the number of mice per group. (P2 = 0.012, Log-rank test; Tri-mAb + IL-2 vs Tri-mAb alone).

All long-term surviving mice following treatment with three doses of Tri-mAb alone or combination with IL-2 were resistant to tumor rechallenge, thereby demonstrating immunological memory against tumor (Figure 5). Interestingly, in one experiment, mice received just one injection of Tri-mAb (with or without IL-2) or three injections of Tri-mAb (with or without IL-2), and when long-term surviving mice from these groups were rechallenged, only mice receiving IL-2 in addition to Tri-mAb completely resisted rechallenge. Mice from the single dose Tri-mAb alone group survived longer than control naïve mice suggesting some degree of resistance, but resistance was only complete in the group receiving coadministration of IL-2 (Figure 5), suggesting that the inclusion of IL-2 could enhance immunological memory formation (P2 = 0.012, Log-rank test; Tri-mAb + IL-2 vs Tri-mAb alone).

### IL-2 increases the frequency of IFN-γ-producing T cells, and CD8+ T cells are necessary for optimal anti-tumor activity

**Figure 6:**
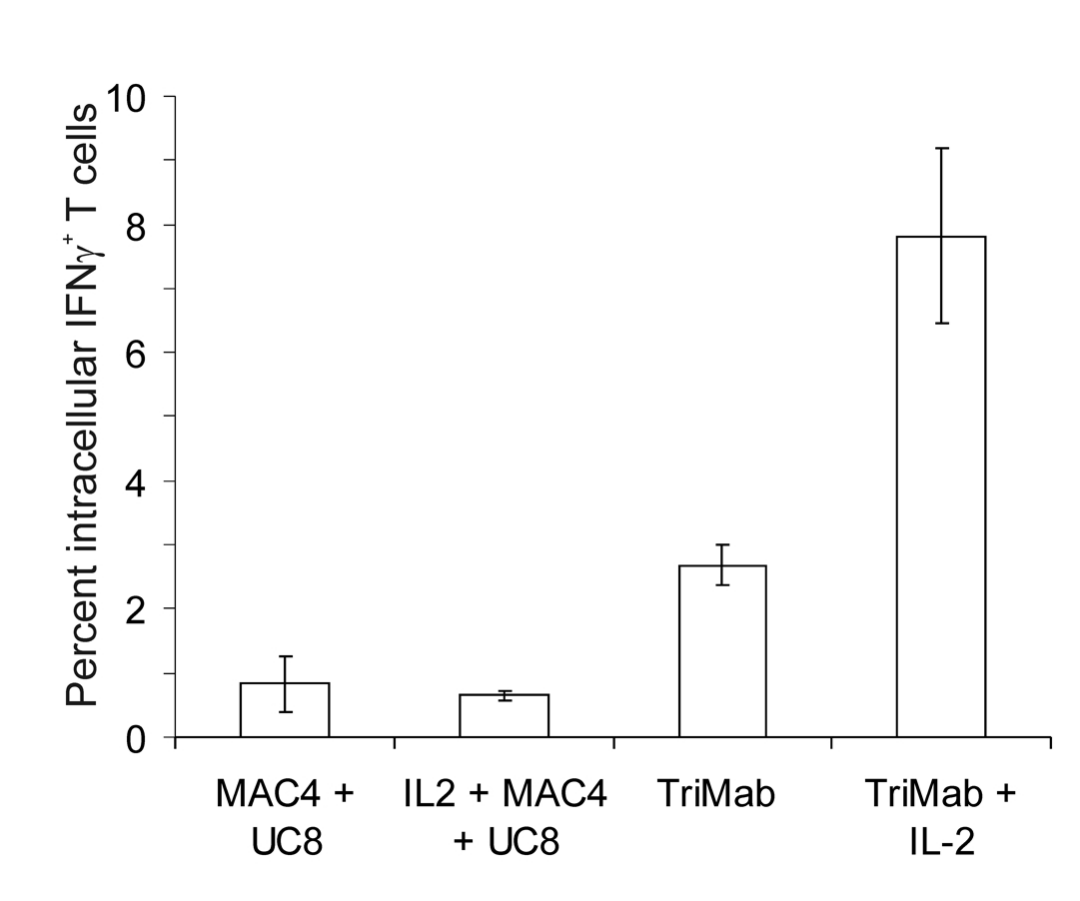
**Coadministration of IL-2 increases the frequency of IFN-γ-producing T cells**. Mice were injected subcutaneously with 2 × 10^5 ^Renca s.c. into the upper foot to enable isolation of popliteal lymph nodes as distinct tumor-draining nodes. Some groups received Tri-mAb i.p. on days 10 and 13, and/or IL-2 at the dose of 10^5 ^IU i.p. on days 10, 11 and 13. Control mice received MAC4 (rat IgG2a) plus UC8-1B9 (hamster IgG) i.p. On day 14, mice were culled and popliteal lymph node cells stimulated on anti-CD3 antibody-coated plates with GolgiStop overnight. Each lymph node analyzed individually. Cells were harvested the next day and stained and assessed by flow cytometry for intracellular IFNγ. Standard error of the mean is shown for 6 mice per group.

To gain insight into the mechanism of the increased anti-tumor activity resulting from inclusion of IL-2 in therapy, we determined the relative proportions of activated T cells in tumor-draining lymph nodes. This was achieved by determining the ability of T cells, freshly isolated from tumor-draining lymph nodes, to produce IFN-γ in response to immobilized anti-CD3. Lymph nodes were taken 4 days after the start of therapy (after 2 doses of Tri-mAb and/or 3 doses of IL-2). While lymph nodes from mice receiving control antibodies or IL-2 alone contained less than 1% IFN-γ-producing T cells, lymph nodes from Tri-mAb-treated mice had a significantly increased proportion of IFN-γ^+ ^T cells (P2 = 0.002) (Figure 6), in agreement with previously published data [13]. However, a further significant increase in the frequency of IFN-γ-producing T cells was afforded by the addition of IL-2 to Tri-mAb therapy (P2 = 0.002) (Figure 6).

**Figure 7:**
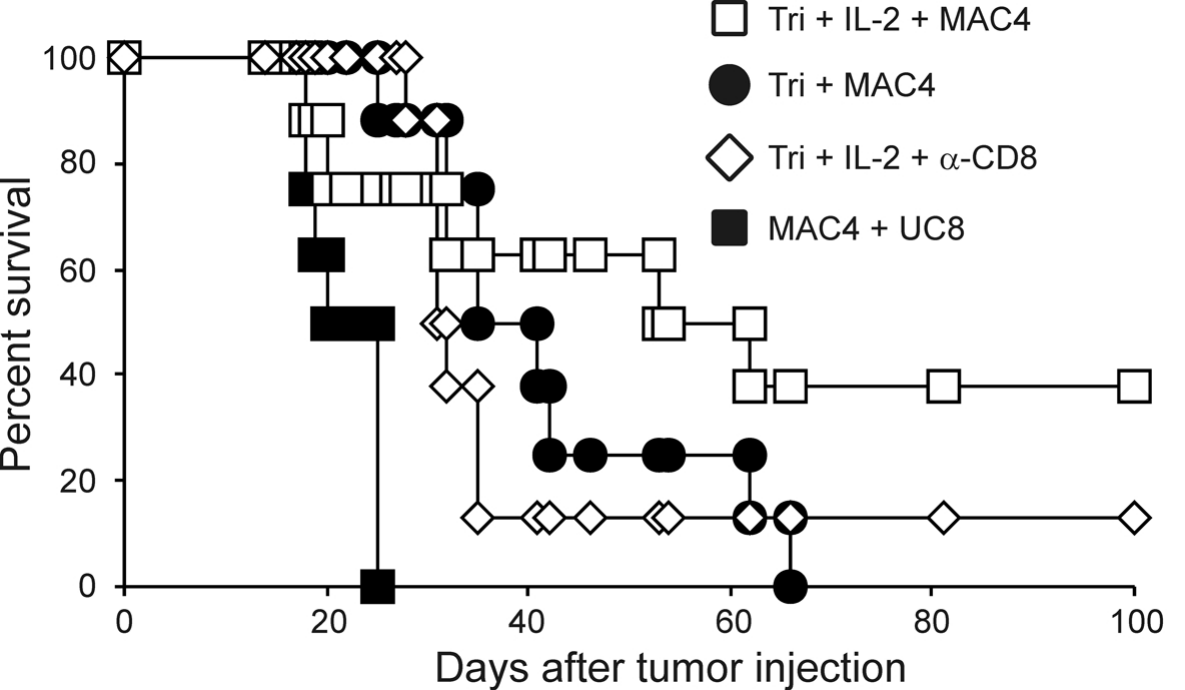
**CD8^+ ^T cells are necessary for optimal anti-tumor effects**. Mice were injected with 1 × 10^5 ^Renca i.k. and then treated with Tri-mAb on day 11 only, and IL-2 at a dose of 10^5 ^IU i.p. on days 11 and 12. Some mice received MAC 4 and UC8-1B9 antibodies as control treatment. In addition, some groups received CD8-depleting mAb as described in Materials and Methods. Results from a single experiment with 8 mice per group, P = 0.035 for Tri-mAb + IL-2 vs CD8-depleted Tri-mAb + IL-2 group (Log-rank test).

To confirm a role for T cells in the observed anti-tumor activity of Tri-mAb + IL-2, cell-depleting antibodies were administered to mice immediately prior to, and during therapy. Since CD4^+ ^regulatory T cells have been shown previously to play a role in tumor progression in this system, we only used anti-CD8 to investigate a role for CD8^+ ^T cells. Depletion of CD8^+ ^T cells largely abrogated the benefit of therapy (Figure 7), suggesting a major role for CD8^+ ^T cells in the therapeutic activity of Tri-mAb + IL-2.

## Discussion

Experimental therapies developed on the basis of responses of ectopic inoculation of tumor cells subcutaneously can be performed quickly and can teach us much about the potential of various therapeutics and give us mechanistic insight into therapeutic effects. Orthotopic metastatic cancer models are more complex and are considered more difficult to treat than subcutaneous tumors. One of the most promising therapies we have recently encountered consists of three agonist antibodies (Tri-mAb) administered systemically to mice bearing subcutaneous DR5^+ ^tumors. However, we did not know if the remarkable tumor eradication rates of approximately 70% could be achieved against orthotopic disease. In experiments described herein, we found that orthotopic kidney cancer was indeed much more difficult to treat than subcutaneous disease, but response rates of mice bearing orthotopic disease could be significantly raised (to ~55%) by the addition of high dose IL-2 to the treatment regimen.

The reasons for poorer responses of orthotopic disease to immunotherapy are not clear, but it is unlikely to simply be an issue of tumor burden, since subcutaneous tumors are larger than the combined mass of primary kidney tumors and micrometastases on day 10 after inoculation, and yet subcutaneous tumors respond better. Possible reasons include differences in tumor microenvironment in which orthotopic disease and metastases have a more immunosuppressive environment perhaps mediated by regulatory cells and/or immunosuppressive cytokines. Another potential reason may lie in inherent differences in lymph nodes draining subcutaneous sites and those draining internal sites. In any event, coadministration of IL-2 was demonstrated to increase the proportion of T cells able to produce IFN-γ upon stimulation, which likely contributed to the enhanced therapeutic effect against orthotopic disease. This may have reflected increases in the frequency or activity of tumor associated antigen-specific T cells, but the specific TAA are not known for Renca, and without this knowledge it is technically difficult to demonstrate tumor-specific activity from freshly isolated lymphoid cells. In any event, it is unlikely that fetal-calf serum antigens played a role in the differential responses since cells injected into both sites were cultured the same, and it is likely fetal calf antigens were not present at the time of treatment since tumors were established for 10 days before treatment.

Tri-mAb therapy is dependent on the expression of DR5 on tumor cells, and ligation of DR5 is thought to induce immunogenic cell death in a proportion of tumor cells, thereby liberating TAA for recognition by the immune system. Nevertheless, it is possible to extend this type of therapy to DR5-refractory tumors by using some chemotherapeutic agents that can also induce immunogenic cell death resulting in enhanced immune responses against tumors when combined with anti-CD40 and anti-CD137 [17,18]. Other orthotopic models of cancer do exist including MC38 mouse colon carcinoma and, although not established in our laboratory, it would be of interest to determine if the current observations extend to these models.

Other combination immunotherapies have demonstrated potential for the eradication of large proportions of established subcutaneous tumors in mice including the use of Toll-like receptor agonists in combination with anti-CD137 [19]. It would be of interest to determine if coadministration of cytokine can enhance therapy against more established subcutaneous disease or orthotopic disease in those settings.

Other forms of therapy have been demonstrated to impact on orthotopic kidney cancer in mice, including the use of IL-2 in combination with anti-CD40 [20] or IL-12 [21] in the Renca system. However, part of the novelty of our observations in the current study lie in the advanced nature of orthotopic disease and the ability to produce a large proportion of long-term survivors that have no evidence of disease upon conclusion of experiments without the need for surgical resection of the primary.

Coadministration of IL-2 has long been known to enhance immunotherapies such as adoptive transfer of tumor-specific T cells [22]. In addition, alternate cytokines can be used with benefit in combination immunotherapies, including IL-15 [23] and IL-21 [15]. Since IL-2 can be associated with toxicity, it would be of interest to determine if these cytokines can be of benefit against orthotopic disease in the current model.

The reasons for the enhancement of therapy for IL-2 are not fully known, although there could be a contribution from increased effector function of T cells mediated by IL-2 [24]. However, IL-2 has also been demonstrated to play a role in the expansion of regulatory T cells [25], and it would be of interest in future studies to determine the effect of IL-2 on regulatory T cell numbers and function in this tumor model. Similarly, we have no insight into the relative contribution of CD4^+ ^helper T cells in this therapy or indeed if they are required at all once IL-2 has been exogenously provided, but it would be of interest to derive such insight in future studies.

There are considerable challenges in the development of optimal immunotherapies for cancer, and it is generally of limited use to stimulate a single immune component, rather it will likely be necessary to use several immune modulators in concert that stimulate diverse immune components such as antigen presentation and T cells, together with activation of innate immune components to produce site-specific inflammatory signals. The choice of appropriate tumor models also represent a challenge, and while subcutaneous models can provide rapid advances and direct further investigations, orthotopic tumor models such as that described herein may provide more physiologic settings in which to determine treatment effectiveness and identify mechanisms. Indeed, the data presented here suggests that the therapeutic efficacy observed in subcutaneous models following immunotherapy may not extend to tumors in other locations. The investigation of rationally designed combination immunotherapies in orthotopic models of malignancy may lead to effective therapeutic options for cancer patients.

## Competing interests

The authors declare that they have no competing interests.

## Authors' contributions

JW contributed to experimental design and participated in all experiments and helped draft the manuscript. PG, SA, PD and HP participated in orthotopic implantation of tumors. JS produced monoclonal antibodies. MS and PD provided crucial reagents and intellectual input in experimental design and interpretation. MK designed and assisted in the execution of experiments and data interpretation, and wrote the manuscript. All authors read and approved the final manuscript.
